# The validity of an area-based method to estimate the size of hard-to-reach populations using satellite images: the example of fishing populations of Lake Victoria

**DOI:** 10.1186/s12982-018-0079-5

**Published:** 2018-08-13

**Authors:** Stephen Nash, Victoria Tittle, Andrew Abaasa, Richard E. Sanya, Gershim Asiki, Christian Holm Hansen, Heiner Grosskurth, Saidi Kapiga, Chris Grundy, Lake Victoria Consortium for Health Research, Pontiano Kaleebu, Andrew Abaasa, Janet Seeley, Freddie Kibengo, Asiki Gershim, Monica Kuteesa, Richard E. Sanya, Alison Elliott, Noah Kiwanuka, Ali Ssetaala, Elizabeth Bukusi, Zachary Kwena, Saidi Kapiga, Christian Hansen, Ramadhan Hashim, Edmund Kisanga, Simon Sichalwe, Heiner Grosskurth, Leslie Nielsen, Jan de de Bont, Anatoli Kamali, Patricia Fast

**Affiliations:** 1 London School of Hygiene and Tropical Medicine, Room G13, Keppel Street, London, WC1E 7HT UK; 2 MRC/UVRI Uganda Research Unit on AIDS, Entebbe, Uganda; 3 Mwanza Interventions Trials Unit (MITU/NIMR), Mwanza, Tanzania

## Abstract

**Background:**

Information on the size of populations is crucial for planning of service and resource allocation to communities in need of health interventions. In resource limited settings, reliable census data are often not available. Using publicly available Google Earth Pro and available local household survey data from fishing communities (FC) on Lake Victoria in Uganda, we compared two simple methods (using average population density) and one simple linear regression model to estimate populations of small rural FC in Uganda. We split the dataset into two sections; one to obtain parameters and one to test the validity of the models.

**Results:**

Out of 66 FC, we were able to estimate populations for 47. There were 16 FC in the test set. The estimates for total population from all three methods were similar, with errors less than 2.2%. Estimates of individual FC populations were more widely discrepant.

**Conclusions:**

In our rural Ugandan setting, it was possible to use a simple area based model to get reasonable estimates of total population. However, there were often large errors in estimates for individual villages.

## Background

Knowing the size of a population is vital to many aspects of public health [1–3] including intervention planning and resource allocation [4]. Population census data are often used to provide this information, but this method is expensive and time-intensive even for small areas [5]. There are also limitations of this method due to population mobility and the time that may have elapsed since a census was conducted [5]. In low-resource settings these limitations are often particularly acute, and so other methods are needed to estimate population sizes [4, 6].

Satellite imagery has been used for population estimates since the 1950s, utilising spectral and spatial satellite data to determine land use for statistical modelling [7]. The use of this technology has grown rapidly since 2005 as high resolution images became widely accessible, increasing access to images of rural settings around the world [3].

There are a number of different ways to estimate the population size of a community from satellite images of the area. One way involves counting structures seen in the images, a method that has been tested in many settings, using both manual counting and automated counts [8]. Comparison of estimates from this method to reference population counts show varying results depending on image quality, particularly regarding their ability to distinguish between individual structures [8, 9]. In locations with clearly visible structures and a reliable estimate of average population per structure for that region, it is possible to estimate population size by simply multiplying the number of structures by the average occupancy per structure. Such estimates, from both manual and automated counts of structures, are typically within a few percent of the results from a survey of the same location [8]. Organisations such as UNHCR, Médecins Sans Frontières, and the International Federation of Red Cross and Red Crescent Societies routinely use this method to estimate size of populations.

The structure-based method is particularly suitable when only a small area is being studied. While crowd-sourcing from groups like Missing Maps or Humanitarian OpenStreetMap Team (HOT) use large numbers of volunteers to cover large land areas [10, 11] there are still inherent problems with this method when image quality is poor or out of date; where individual structures cannot be seen; or where the mobilisation of sufficient numbers of volunteers is a challenge.

An alternative approach is to use the area of a village, rather than the number of buildings. Estimating the area of a village doesn’t rely on such high resolution satellite images as is required to count individual structures, and is also quicker to do using straightforward geographical software. Hence, if a reliable estimate of population density was available for a particular geographical region, it is possible to estimate population size simply by multiplying this population density by the area of the region of interest.

The aim of this paper is to establish if there is indeed an average population density figure which can be used to produce reasonable estimates of population size for rural villages. We will use known population data from fishing communities on or near the shores of Lake Victoria in Uganda. We will use two thirds of the villages to produce an estimate of population density, which we will then use to produce population estimates for the remaining villages. We will also present the overall population density of all villages, in the hope that this figure can be used and tested by other researchers working in East Africa. An open question is in which locations and settings will it be reasonable to use this average population density to estimate populations.

We also explore whether the simple model of “density times area” described above works as well as more complicated, but still straightforward to implement, regression models. We investigate if these methods provide reasonable population estimates of both the overall area and of individual villages.

Our hope is that the methods explored here will allow a reasonable population estimate to be produced at low cost in terms of time and resources, and that the estimates will be of sufficient accuracy for use in situations where census data are unavailable.

## Methods

### Setting

We use data from the fishing communities of Lake Victoria in Uganda. The villages were selected as already having been surveyed in previous research by the research teams from the MRC/UVRI Uganda Research Unit and therefore accurate population data and global positioning system (GPS) location for each village were available. All estimates obtained from the methods described below were compared to these ground survey data. A fishing community (FC) was defined as a residential area in which the majority of the residents rely on Lake Victoria for income generation. Household surveys were conducted in 2012–13 and counted number of households and number of people in each household [12, 13]. All of the villages are fishing communities, with 39 on the mainland and 27 on the islands of Lake Victoria. These communities are characterised by single storey buildings, with the majority used for residential purposes. These communities are hard-to-reach, poorly served by skilled health care providers and have poor access to clean water and sanitation. Health issues include HIV, helminth infection, malaria, and high maternal and newborn morbidity. The populations of these communities are typically very mobile, consisting of transient populations who move between villages and within the wider region and country.

Each community was viewed in Google Earth Pro software (GEP) and communities with no central cluster of residential structures were excluded. We also excluded fishing communities for which GPS coordinates did not show up as a village on the available satellite imagery, or where satellite images were unavailable.

### Estimation of area using GEP

**Fig. 1:**
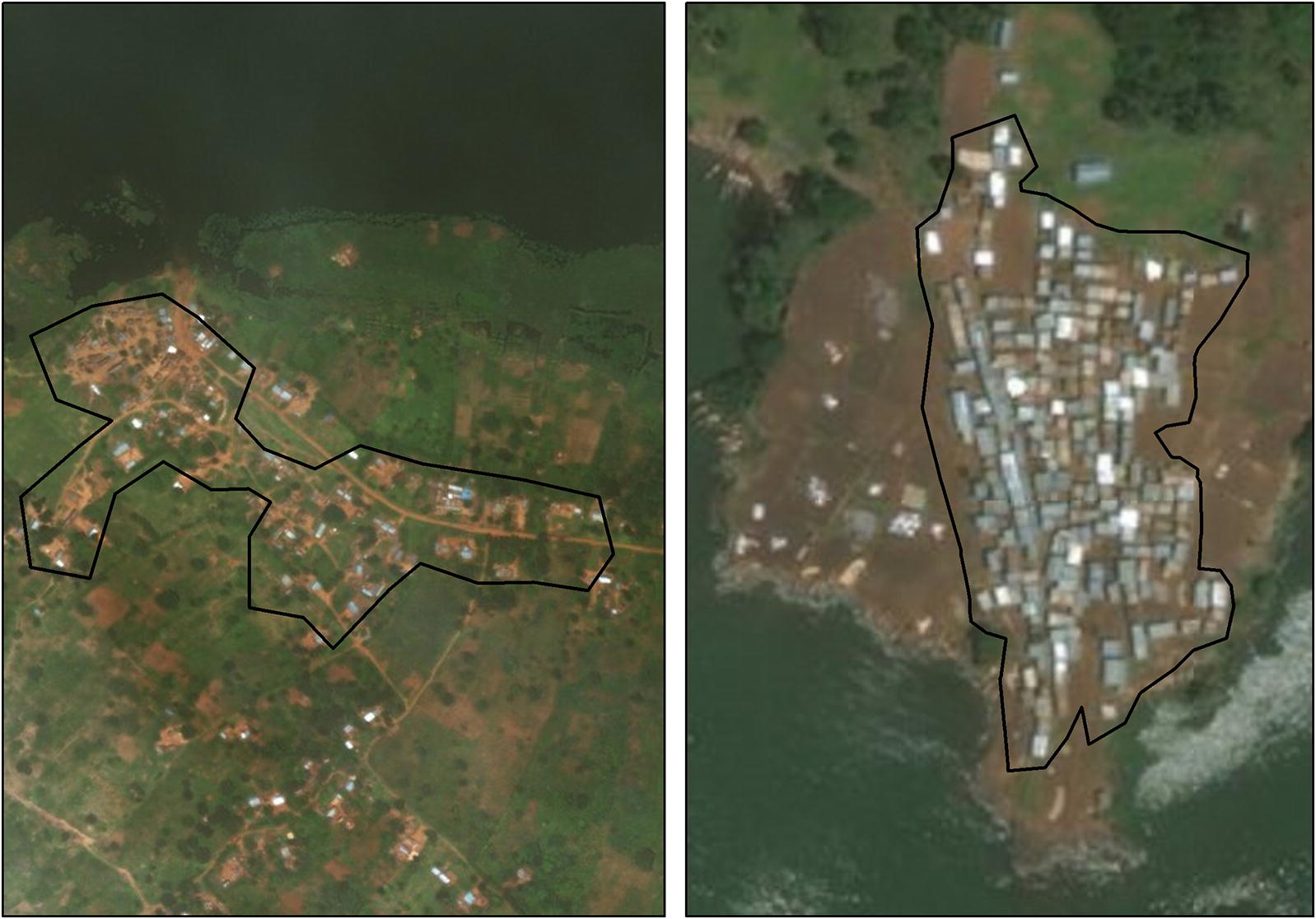
Examples of boundaries fitted to the typical satellite images of FC

For each fishing community with satellite imagery available, we used GEP software to assess the area as follows. A member of our team [CG] estimated the perimeter of each community based on where structures were observable, and assessed density as either low or high, based on the space visible between structures on the satellite image (see Fig. 1). Although the perimeter was drawn so as to enclose the majority of structures which naturally formed the community, it was occasionally the case that some structures were excluded. The area enclosed within the perimeter was calculated automatically by the GEP software. We estimate that this process took less than 1 min per FC.

### Estimation of populations

We compared three methods of estimating populations: two using the average density and one using a regression model. The two average density methods calculated the average in different ways: the first used the average of the individual FC densities; the second used the overall population density calculated by summing the population of all FCs and dividing by the total area. We refer to these two methods as AD1 and AD2. The simple linear regression model we used consisted of a constant term and the FC area as the single predictor. The average density methods can be considered as regression models without a constant term; this allows the first two methods to be described as:

1 $${\text{Y}}_{\text{i}} =\upbeta \times {\text{area}}_{\text{i}}$$

where Y_i_ is the predicted population for village i, and β is the average population density (however calculated). The regression method can be described as

2 $${\text{Y}}_{\text{i}} =\upalpha^{*} +\upbeta^{*} \times {\text{area}}_{\text{i}}$$

where α* and β* are the regression coefficients representing the intercept and slope respectively. All population estimates are presented rounded to the nearest whole number; when calculating total populations by summing individual populations the original estimates were used.

To allow us to test and compare these approaches we randomly split the data into two sets: an index set of 31 FCs which we used to calculate the parameters (average density and regression coefficients) and a test set of 16 FCs which we used to compare the predictions made by these parameters with the values from the earlier surveys. We also calculated the unstratified parameters in the entire dataset of 47 FC’s, as these are the best available estimates from the data we have. We report each of these parameters with a 95% confidence interval (CI), with the exception of the M2 parameter for which a CI cannot be calculated as it is the simple ratio of total population to total area.

### Average density methods

To calculate the average density of FC for M1 we first calculated the density in each of the 31 index FCs and then used the mean of these figures as the parameter β_M1_. We then applied this value to each of the 16 test FCs to predict their population, and summed these estimates to give a total population for the test FCs. For M2 we calculated the total population of the 31 index communities and divided by their total area, and again used this parameter β_M2_ to calculate the populations of the remaining FC.

### Regression method

We ran a simple linear regression, using area as a predictor of population, on the 31 index FCs. We took the parameters from this regression (α*, the intercept and β*, the coefficient for area) applied them to the 16 test FCs. We summed these individual estimates to get an estimate for the total population of the 16 test FCs. Note that because the constant is calculated at the village level, it was not possible to apply these parameters to an entire region; they must be applied at the village level.

### Stratifying by location and density

We repeated the above twice: once stratifying on location (island/mainland) and once stratified on assessed density category (low/high). In each case, we used the same original set of index and test FC, to enable comparison between the methods. We then separately calculated parameters in each stratum, and applied them to the test FC according to stratification level. This is equivalent to allowing an interaction between area and the stratification factor in Eqs. 1 and 2; alternatively it can simply be expressed as separate equations with equivalent parameters for each level of the stratification factor. That is, parameters β_island_, β_mainland_, β_low-density_, and β_high-density_, and similarly for β*, α and α*.

Stata v15.0 was used for population estimation and GEP was used to obtain satellite images and estimate areas.

## Results

We had population data on 66 FCs. Of these, eight were excluded due to absence of satellite imagery, nine due to no village being visible on the satellite image and one due to no GPS coordinate for the population data.

**Fig. 2:**
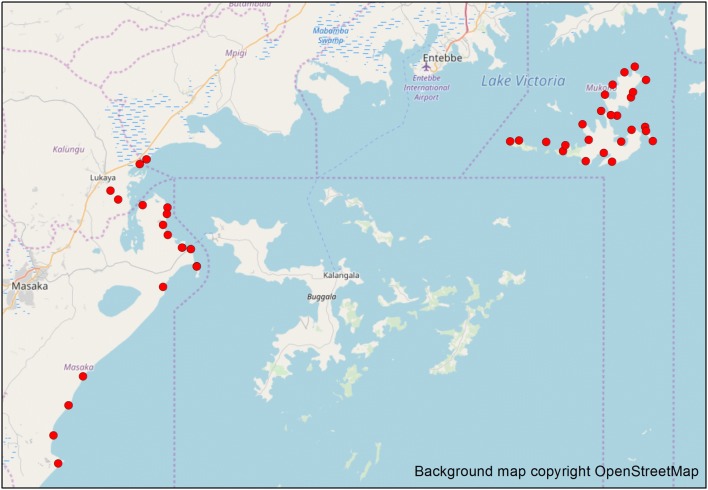
Map of Uganda and Lake Victoria. Red dots indicate location of FCs used in this study

**Fig. 3:**
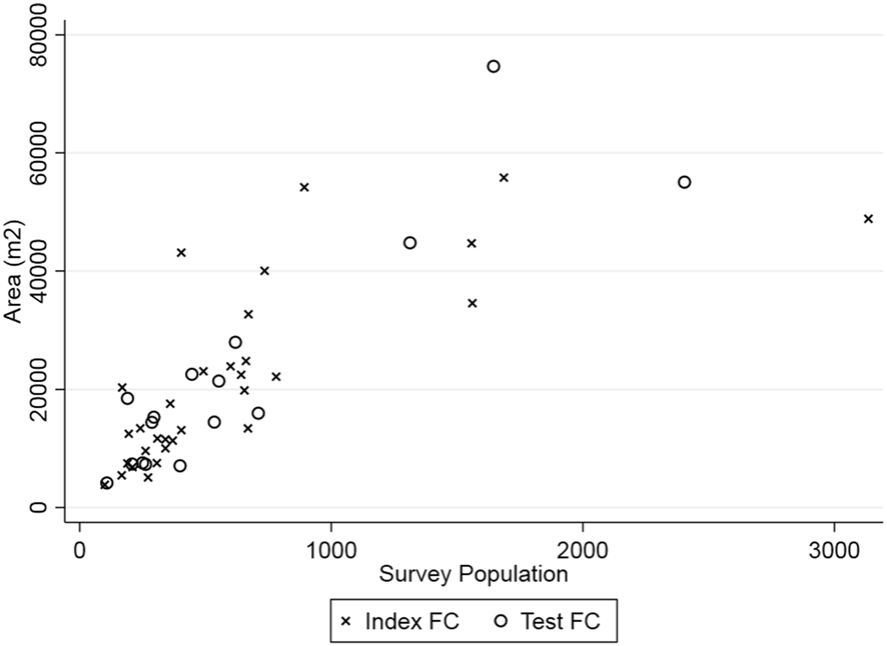
Plot of area against survey population for each FC

The analysis presented here was conducted on 47 FCs (see map, Fig. 2). The total population from surveys was 29,574. The combined area was 1,030,918 m^2^, giving an overall average population density of 0.0293 people per m^2^. The mean of the individual densities across the 47 communities was 0.0287 people per m^2^. The mean FC population was 629, but there was large variation among the individual communities (Table 1). The range in FC population was from 99 to 3134, with a standard deviation of 616. The population density ranged from 0.008 to 0.064 per m^2^, with a standard deviation (SD) of 0.012. Twenty-six communities were situated on islands; with the remainder on the mainland. The mean of individual population densities were 0.0319 and 0.0260 per m^2^ for island and mainland FC respectively. The overall average population density was 0.0306 on islands and 0.0270 on the mainland. A plot of area against survey population is shown in Fig. 3.

**Table 1:** Characteristics of fishing communities

Id	Name	Mainland	Assessed density	Population	Area (m^2^)	Density	Index	Date of survey	Date of image
1	Kibanga	Island	Low	189	7517	0.0251	Index	October 2012–July 2013	11/10/2014
2	Bbaale	Island	Low	240	13,456	0.0178	Index	October 2012–July 2013	07/07/2013
3	Zingoola	Island	Low	670	32,696	0.0205	Index	October 2012–July 2013	11/10/2014
4	Busi	Island	High	166	5502	0.0302	Index	October 2012–July 2013	11/10/2014
5	Busiro	Island	High	209	6880	0.0304	Index	October 2012–July 2013	27/02/2015
6	Muga-Ngogo	Island	High	261	9635	0.0271	Index	October 2012–July 2013	11/10/2014
7	Tabaliro	Island	High	271	5144	0.0527	Index	October 2012–July 2013	08/01/2015
8	Nambu	Island	High	308	11,726	0.0263	Index	October 2012–July 2013	27/02/2015
9	Katooke	Island	High	340	11,588	0.0293	Index	October 2012–July 2013	27/02/2015
10	Kachanga	Island	High	403	13,151	0.0306	Index	October 2012–July 2013	27/02/2015
11	Lubembe	Island	High	641	22,531	0.0284	Index	October 2012–July 2013	11/10/2014
12	Mwoma	Island	High	654	19,883	0.0329	Index	October 2012–July 2013	11/10/2014
13	Kitosi	Island	High	660	24,861	0.0265	Index	October 2012–July 2013	11/10/2014
14	Kakeeka	Island	High	668	13,441	0.0497	Index	October 2012–July 2013	11/10/2014
15	Misenyi	Island	High	734	40,065	0.0183	Index	October 2012–July 2013	11/10/2014
16	Myenda	Island	High	780	22,208	0.0351	Index	October 2012–July 2013	11/10/2014
17	Kiimi	Island	High	1557	44,725	0.0348	Index	October 2012–July 2013	11/10/2014
18	Kalyambuzi	Island	High	1560	34,577	0.0451	Index	October 2012–July 2013	27/02/2015
19	Kansambwe	Island	High	1685	55,858	0.0302	Index	October 2012–July 2013	08/01/2015
20	Buzirango	Mainland	Low	99	3910	0.0253	Index	March 2013–May 2013	17/01/2012
21	Kamaliba	Mainland	Low	168	20,371	0.0082	Index	March 2013–May 2013	13/08/2012
22	Bbale	Mainland	Low	194	12,539	0.0155	Index	March 2013–May 2013	17/01/2012
23	Kabasese	Mainland	Low	340	10,060	0.0338	Index	March 2013–May 2013	17/01/2012
24	Mitondo	Mainland	Low	359	17,639	0.0204	Index	March 2013–May 2013	17/01/2012
25	Kamunga	Mainland	Low	403	43,141	0.0093	Index	March 2013–May 2013	13/08/2012
26	Kachanga	Mainland	Low	491	23,123	0.0212	Index	March 2013–May 2013	25/11/2008
27	Kaziru	Mainland	Low	892	54,227	0.0164	Index	March 2013–May 2013	17/01/2012
28	Makonzi	Mainland	High	306	7572	0.0404	Index	March 2013–May 2013	17/01/2012
29	Kabasese	Mainland	High	369	11,376	0.0324	Index	March 2013–May 2013	13/08/2012
30	Kachanga	Mainland	High	599	23,957	0.0250	Index	March 2013–May 2013	17/01/2012
31	Lambu	Mainland	High	3134	48,875	0.0641	Index	March 2013–May 2013	25/11/2008
32	Lwanga Muto	Island	Low	208	7367	0.0282	Test	October 2012–July 2013	11/10/2014
33	Kisigala	Island	Low	535	14,474	0.0370	Test	October 2012–July 2013	11/10/2014
34	Maala	Island	High	250	7564	0.0331	Test	October 2012–July 2013	02/02/2015
35	Kayunyu	Island	High	287	14,439	0.0199	Test	October 2012–July 2013	11/10/2014
36	Batwala	Island	High	399	7072	0.0564	Test	October 2012–July 2013	27/01/2015
37	Lugumba	Island	High	446	22,574	0.0198	Test	October 2012–July 2013	11/10/2014
38	Kisso	Island	High	710	15,986	0.0444	Test	October 2012–July 2013	27/02/2015
39	Kalangala	Mainland	Low	108	4167	0.0259	Test	March 2013–May 2013	17/01/2012
40	Kamaliba	Mainland	Low	190	18,497	0.0103	Test	March 2013–May 2013	13/08/2012
41	Kassa	Mainland	Low	262	7316	0.0358	Test	March 2013–May 2013	17/01/2012
42	Kalokoso	Mainland	Low	295	15,313	0.0193	Test	March 2013–May 2013	07/07/2013
43	Kisuku	Mainland	Low	553	21,424	0.0258	Test	March 2013–May 2013	17/01/2012
44	Namirembe	Mainland	Low	619	28,004	0.0221	Test	March 2013–May 2013	22/12/2009
45	Kamuwunga	Mainland	Low	1313	44,771	0.0293	Test	March 2013–May 2013	13/08/2012
46	Ddimo	Mainland	Low	1645	74,668	0.0220	Test	March 2013–May 2013	07/07/2013
47	Lambu	Mainland	High	2404	55,048	0.0437	Test	March 2013–May 2013	25/11/2008

We assessed 21 FCs as low density and 26 as high density. Of the 21 assessed as low density, the actual density calculated from the survey population ranged from 0.008 to 0.037, with a mean of 0.0224 per m^2^. The overall average population density was 0.0206 per m^2^. For those FCs assessed as high density, the actual density ranged from 0.018 to 0.064 with a mean of 0.0348. The overall average population density was 0.0356 per m^2^. Of the 21 FCs with lowest actual density, we assessed 16 (correctly) as low density and five (incorrectly) as high density. One possible explanation for this misclassification is the amount of time between the survey and the satellite image.

The 31 FCs assigned to the index set had an overall population of 19350 and a total area of 672234 m^2^. The equivalent figures for the 16 test FCs were 10224 and 358684 m^2^.

### Parameter estimates

The average density of the 31 index FCs was β_M1_ = 0.0291 per m^2^. The overall average population density was β_M2_ = 0.0288. The regression parameters were β* = 0.0302 for the slope and α* = − 30.36 for the constant. In the entire dataset of 47 FC’s the M1 parameter was 0.0293, with a 95% CI of (0.025, 0.0328). The M2 parameter was 0.0287. The regression parameters in the complete dataset were α = − 13.32, with a 95% CI of (− 198.86, 172.22) and β = 0.0293, 95% CI (0.0225, 0.0361). All other regression parameters are shown in Table 2.

**Table 2:** Parameters from estimation methods

Method	M1	M2	Regression	
Parameter	Beta	Beta	Alpha	Beta
All FC’s	0.0293	0.0287	− 13.3193	0.0293
95% CI	(0.0258, 0.0328)	–	(− 198.86, 172.22)	(0.0225, 0.0361)
Index FC’s only				
All index FC’s	0.0291	0.0288	− 30.3628	0.0302
Low density	0.0194	0.0194	86.8760	0.0129
High density	0.0345	0.0345	− 93.6135	0.0396
Island	0.0311	0.0303	16.5572	0.0295
Mainland	0.0260	0.0266	− 112.4567	0.0314

### Total population

The average density method M1 predicted a total population of the 16 test FC of 10452, an overestimate of 228 (2.2%) compared to the census data. Method M2 predicted a total population of 10325, also an overestimate, of 101 (0.99%). The regression method predicted a total population of 10341, an overestimate of 117 (1.1%).

### Individual FC populations

Using method M1, the largest absolute discrepancy of an individual FC population was 800 (FC #47; population 2404; estimated population 1604). The same FC also produced the largest error from the other two methods: M2 predicted 1585 (discrepancy = 819) and the regression method predicted 1631 (discrepancy = 773). The largest relative discrepancy for all three methods was with the FC #40, which has a survey population of 190. Using M1, our prediction was 539 (83.7% overestimate), using M2 it was 532 (80.0% overestimate) and using the regression method it was 528 (77.9% overestimate). Full results are shown in Table 3.

**Table 3:** Estimates of individual FC populations from all methods

Id	Name	Population estimates			Discrepancy			% discrepancy		
		M1	M2	Reg	M1	M2	Reg	M1	M2	Reg
32	Lwanga Muto	215	212	192	7	4	− 16	3.37	1.92	− 7.69
33	Kisigala	422	417	407	− 113	− 118	− 128	− 21.12	− 22.06	− 23.93
34	Maala	220	218	198	− 30	− 32	− 52	− 12.00	− 12.80	− 20.80
35	Kayunyu	421	416	405	134	129	118	46.69	44.95	41.11
36	Batwala	206	204	183	− 193	− 195	− 216	− 48.37	− 48.87	− 54.14
37	Lugumba	658	650	651	212	204	205	47.53	45.74	45.96
38	Kisso	466	460	452	− 244	− 250	− 258	− 34.37	− 35.21	− 36.34
39	Kalangala	121	120	95	13	12	− 13	12.04	11.11	− 12.04
40	Kamaliba	539	532	528	349	342	338	183.68	180.00	177.89
41	Kassa	213	211	190	− 49	− 51	− 72	− 18.70	− 19.47	− 27.48
42	Kalokoso	446	441	432	151	146	137	51.19	49.49	46.44
43	Kisuku	624	617	616	71	64	63	12.84	11.57	11.39
44	Namirembe	816	806	815	197	187	196	31.83	30.21	31.66
45	Kamuwunga	1305	1289	1321	− 8	− 24	8	− 0.61	− 1.83	0.61
46	Ddimo	2176	2149	2223	531	504	578	32.28	30.64	35.14
47	Lambu	1604	1585	1631	− 800	− 819	− 773	− 33.28	− 34.07	− 32.15

*Reg* regression

### Stratification by location

Stratifying by location did not improve the overall estimates of the average density methods, but did improve the regression method. The estimated overall population using M1 was 9788, an underestimate of 436 (4.3%); using M2 the estimate was 9867, an underestimate of 357 (3.5%); and using the regression method the estimate was 10212, an underestimate of just 0.12%. Full results for individual FC population estimates are shown in Table 4. The largest discrepancies again occurred in predictions of FC #47 (absolute error) and FC #40 (relative error).

**Table 4:** Population estimates from analysis stratified by location

Id	Name	Population estimates			Discrepancy			% discrepancy		
		M1	M2	Reg	M1	M2	Reg	M1	M2	Reg
32	Lwanga Muto	229	223	234	21	15	26	10.10	7.21	12.50
33	Kisigala	450	439	444	− 85	− 96	− 91	− 15.89	− 17.94	− 17.01
34	Maala	235	229	240	− 15	− 21	− 10	− 6.00	− 8.40	− 4.00
35	Kayunyu	449	438	443	162	151	156	56.45	52.61	54.36
36	Batwala	220	215	225	− 179	− 184	− 174	− 44.86	− 46.12	− 43.61
37	Lugumba	702	685	683	256	239	237	57.40	53.59	53.14
38	Kisso	497	485	489	− 213	− 225	− 221	− 30.00	− 31.69	− 31.13
39	Kalangala	108	111	19	0	3	− 89	0.00	2.78	− 82.41
40	Kamaliba	481	491	469	291	301	279	153.16	158.42	146.84
41	Kassa	190	194	118	− 72	− 68	− 144	− 27.48	− 25.95	− 54.96
42	Kalokoso	398	407	369	103	112	74	34.92	37.97	25.08
43	Kisuku	557	569	561	4	16	8	0.72	2.89	1.45
44	Namirembe	729	744	768	110	125	149	17.77	20.19	24.07
45	Kamuwunga	1165	1190	1295	− 148	− 123	− 18	− 11.27	− 9.37	− 1.37
46	Ddimo	1943	1984	2235	298	339	590	18.12	20.61	35.87
47	Lambu	1432	1463	1618	− 972	− 941	− 786	− 40.43	− 39.14	− 32.70

*Reg* regression

### Stratifying by assessed density category

Using method M1 stratified by assessed density, the overall population estimate was 8815, compared to a survey population of 10224. This is an underestimate of 1409 (13.8%). Using method M2 produced an estimate of 8330, an underestimate of 1894 (18.5%); the regression method fared worse with a population estimate of 8222, an underestimate of 2002 (19.6%). Full results are shown in Table 5.

**Table 5:** Population estimates from analysis stratified by assessed population density

Id	Name	Population estimates			Discrepancies			% discrepancies		
		M1	M2	Reg	M1	M2	Reg	M1	M2	Reg
32	Lwanga Muto	143	125	182	− 65	− 83	− 26	− 31.25	− 39.90	− 12.50
33	Kisigala	281	245	274	− 254	− 290	− 261	− 47.48	− 54.21	− 48.79
34	Maala	261	267	206	11	17	− 44	4.40	6.80	− 17.60
35	Kayunyu	498	510	478	211	223	191	73.52	77.70	66.55
36	Batwala	244	250	187	− 155	− 149	− 212	− 38.85	− 37.34	− 53.13
37	Lugumba	778	797	801	332	351	355	74.44	78.70	79.60
38	Kisso	551	564	540	− 159	− 146	− 170	− 22.39	− 20.56	− 23.94
39	Kalangala	81	71	141	− 27	− 37	33	− 25.00	− 34.26	30.56
40	Kamaliba	359	313	326	169	123	136	88.95	64.74	71.58
41	Kassa	142	124	182	− 120	− 138	− 80	− 45.80	− 52.67	− 30.53
42	Kalokoso	297	260	285	2	− 35	− 10	0.68	− 11.86	− 3.39
43	Kisuku	416	363	364	− 137	− 190	− 189	− 24.77	− 34.36	− 34.18
44	Namirembe	544	475	449	− 75	− 144	− 170	− 12.12	− 23.26	− 27.46
45	Kamuwunga	870	759	666	− 443	− 554	− 647	− 33.74	− 42.19	− 49.28
46	Ddimo	1450	1265	1053	− 195	− 380	− 592	− 11.85	− 23.10	− 35.99
47	Lambu	1898	1943	2087	− 506	− 461	− 317	− 21.05	− 19.18	− 13.19

*Reg* regression

## Discussion

The simple area-based methods described here produced reasonable estimates of the overall population of our test communities, which seem to us to be sufficiently accurate for many situations, particularly where formal census data are unavailable. For example, when estimating public health needs for a group of villages, this approximate population estimate would be sufficient. The most accurate method for predicting the overall population used a simple average population density. Stratifying by location or assessed density did not improve the estimates; using density was particularly unsuccessful, possibly due to the difficulty in correctly categorising communities from satellite imagery. Using a regression approach did not increase accuracy. We therefore recommend the simplest, average density approach. This has the advantage of being intuitive, easy to understand and calculate, and doesn’t require village-level data calculations. This method requires just one parameter and knowledge of the combined area of all the communities in which the population is to be estimated. In our data the average density parameter was 0.0287 per m^2^. It is an open question if this figure is applicable to other geographical regions or other types of settings, for example, mining communities or other small isolated villages. We plan to test the applicability of this method, and this average density figure, in future work using population data from surveys of other regions of East Africa.

All methods were less successful in predicting the populations of individual settlements. This is of no great surprise, given heterogeneity of densities observed; the most heavily populated FC was almost eight times as dense as the least. We would therefore not advise using these methods to predict the populations of individual communities.

For area-based methods to work there is an assumption that the villages are “similar”, that is, the villages you wish to estimate the population of have a similar population density to those which provided our figure of 0.0287. We recognise that the way we have designed this study guarantees that this requirement will be satisfied. However, we hope that this may be applicable in other communities around Lake Victoria, or fishing communities based on other lakes in East Africa. Area based methods have historically been used in urban environments [14, 15] or used large numbers of variables to define typologies [16]. Large amounts of the work around defining populations through remotely sensed data is in defining these typologies.

Small villages in many parts of rural Africa consist of buildings of similar type: single-storey, with little variation in building materials and construction. It is therefore surprising to find the variation in population density in FCs as we observed around the Ugandan shores of Lake Victoria. The extremes of variation may be due to the different types of FCs, some being villages that are on the lakeshore and so fishing is their main livelihood, while others may be temporary and used during certain parts of the year when the fish are in that location. The communities may be more cramped due to space limitations on islands or peninsular areas. There could also be a difference in the population makeup, as fishing communities often consist primarily of working age men and contain fewer families, resulting in varying average building occupancy rates. These would both alter the population density of the FC. We also acknowledge the difference between the date of the survey and the date the corresponding satellite image was taken; this could increase errors in our predictions if there were significant changes in the FC population between those dates.

Following our analysis, we applied parameters from our complete dataset to data from 509 FC villages in Uganda, identified in GEP, for which we had area, but not population data. The total area was 10,946,521 m^2^. The average density method M1 estimated an overall population of 320,543; method M2 estimated 314023; and the regression method estimated 313,891. Whilst it is impossible to verify the accuracy of these estimates, it is reassuring that in this setting they produced similar values and gives us hope that the simplest method is not significantly worse than a more complex approach even when estimating larger populations in a large number of villages.

The use of GEP as the source of images and the method to define area had pros and cons. It is easy to use and readily available. These are great advantages for groups in which there is little or no Geographic Information System (GIS) experience. In a very short time, it is possible to learn how to mark regions and to extract the area. Training carried out with researchers from the three countries which border Lake Victoria gave us first-hand experience that GEP could be learned in less than one hour. It was also very quick to map areas: the dataset for all villages along the Ugandan lakeshore of Lake Victoria took one person less than three to produce.

The availability and age of imagery is more of an issue. In some areas, images are plentiful and are often taken many times per year. These areas are typically areas of human activity (cities, areas of conflict, deforestation) or where natural disasters have occurred. But this is not the case in all parts of the world, particularly in more rural areas. Around Lake Victoria most images happened to be from 2012, the preceding 12 months to the majority of the population surveys. There was often only one image available. Although images were available for the majority of the area, these were not always the very high sub-metre resolution images best suited to assessing structures, but did allow the populated area to be identified.

In communities that are very stable with little change the image date is not an issue. However, in fishing communities such as those in this research, change can be very rapid, in terms of both increase and decrease in size. If the community is temporary (for instance the duration of a fishing season) then the residents may move sites frequently. There is a similarity with displaced populations, which also can change rapidly. A further limitation is that we excluded structures which were located away from the main village. This was done to ease the process of defining the boundary, and hence obtain an estimate for the village area. However, it does mean that if there are varying numbers of people living in structures away from a village, this method may not be appropriate.

The date and availability of images resulted in us having to exclude 19 villages from the analysis. In addition, the calculation of FC area from satellite images may have been inaccurate. One advantage of a simple area method is that the village areas do not have to come from satellite images; it would be possible for fieldworkers to use a handheld GPS device to define the outline of the village and thus calculate the area, removing these inaccuracies.

Our results match what is typical of other studies [7], that errors are larger for individual areas than for the population as a whole. Using a simple area method to estimate populations of groups of villages is feasible and would be a rapid and low skills way to get populations in these settings. Care would be needed to use this method to estimate populations in individual villages. Further work is needed to investigate if assigning typologies, or using more recent satellite images (or calculating area from on the ground) would improve the results.

Some progress has recently been made by organisations on population datasets such as WorldPop (Stevens FR). These datasets are still far from complete and could not be used around Lake Victoria in our target villages. There is an overlap in the work of defining population densities for different urban typologies, and it is important that data from small surveys are able to feed into the large datasets and vice versa. There is also a need for the larger datasets to be made available through simple-to-use software, and not rely so heavily on GIS skills. However it is filled, there is still a gap for a rapid, low skill method that can be applied in settings where GIS capabilities are very limited or population change was rapid.

## Conclusion

Simplified methods are needed to determine the size of populations at high health risk in resource-limited settings. Satellite images may be able to help provide information in areas where access and resources to perform surveys is limited, or for which a rapid estimation is required. We have shown that it is straightforward to generate the required spatial data using widely available software such as GEP, without the need for more technical GIS skills. We have shown that using an average density of 0.0287 per m^2^ provides a reasonable estimate of population for a group of communities. However, care is needed when using area based methods with migratory populations, where estimates for individual communities may be associated with large errors. Overall population estimations balance out, and with further validation in more stable communities it may prove to be more viable for individual locations. Similarly using a GPS device to obtain the area of the village and multiplying by regional population densities would give a simple method where visiting the location was an option.
